# Laparoscopic Radical Colectomy with Complete Mesocolic Excision Offers Similar Results Compared with Open Surgery

**DOI:** 10.3390/medicina61071231

**Published:** 2025-07-07

**Authors:** Vasile V. Bintintan, Vlad Fagarasan, Radu I. Seicean, David Andras, Alexandru I. Ene, Romeo Chira, Adriana Bintintan, Georgiana Nagy, Cristina Petrisor, Simona Cocu, Elena Stefanescu, Ionut Negoi, Adrian Calborean, George C. Dindelegan, Ciprian Silaghi, Iulia Lupan, Gabriel Samasca

**Affiliations:** 1 1st Surgical Clinic, “Iuliu Hatieganu” University of Medicine and Pharmacy, 400006 Cluj-Napoca, Romania; vasile.bintintan@umfcluj.ro (V.V.B.); vlad.fagarasan@yahoo.com (V.F.); rseicean@yahoo.com (R.I.S.); andrasdavid88@gmail.com (D.A.); ilieene.alexandru@gmail.com (A.I.E.); george.dindelegan@gmail.com (G.C.D.); 2 1st Medical Clinic, “Iuliu Hatieganu” University of Medicine and Pharmacy, 400006 Cluj-Napoca, Romania; romeochira@yahoo.com (R.C.); abintintan@yahoo.com (A.B.); georgi_nagy@yahoo.com (G.N.); 3 2nd Intensive Care Unit Department, “Iuliu Hatieganu” University of Medicine and Pharmacy, 400006 Cluj-Napoca, Romania; petrisor.cristina@umfcluj.ro (C.P.); simonacocu2@gmail.com (S.C.); stefanescuelena2004@yahoo.com (E.S.); 4 Emergency Hospital of Bucharest, “Carol Davila” University of Medicine and Pharmacy, 010271 Bucharest, Romania; negoiionut@gmail.com; 5 National Institute for Research and Development of Isotopic and Molecular Technologies, 400293 Cluj-Napoca, Romania; adrian.calborean@gmail.com; 6 Department of Medical Biochemistry, “Iuliu Hatieganu” University of Medicine and Pharmacy, 400349 Cluj-Napoca, Romania; 7 Interdisciplinary Research Institute on Bio-Nano-Sciences, Babes Bolyai University, 400271 Cluj-Napoca, Romania; iulia.lupan@ubbcluj.ro; 8 Department of Immunology, University of Medicine and Pharmacy, 400006 Cluj-Napoca, Romania; gabriel.samasca@umfcluj.ro

**Keywords:** complete mesocolic excision, laparoscopic colonic resection, colonic cancer, medial-to-lateral dissection, Toldt fascia

## Abstract

*Background and Objectives*: The technique of complete mesocolic excision (CME) for colonic cancer is being advocated to improve the local control of the disease and increase the long-term survival. However, even with an open approach, CME is a complex technique and has not yet been adopted as standard care. Laparoscopy has been proven to bring significant advantages to colorectal surgery but performing a laparoscopic CME (Lap-CME) for colonic cancer is even more technically demanding than CME in open surgery. The purpose of this study is to evaluate whether Lap-CME can be offered as a standard procedure for patients with colonic cancer and to compare the results with those obtained after a conventional, open technique. *Materials and methods*: This study included 100 consecutive patients with colonic cancer, who were operated on by the same surgical team using a standardized medial-to-lateral open or laparoscopic complete mesocolic excision technique. The perioperative data was prospectively recorded in a database and retrospectively analyzed with the aim of identifying the proportion of patients that received Lap-CME, to evaluate the success rate of the procedure and to identify whether there are differences in the oncological quality of CME between the laparoscopic and open surgery groups. *Results*: Most of the patients enrolled in this study were in the advanced stages of the disease, with the incidence of pT3 tumors being 67% and the mean tumor size averaging 4.5 cm. Laparoscopic CME was performed in 39% of cases overall, with 41.4% being right colectomies, 42.5% being left colectomies and 16.1% being transverse colectomies. All of the parameters relevant to the oncological quality of resection, namely total lymph node count, resection margins, or the completeness of resection, were similar between the open and laparoscopic groups both when analyzed for the entire cohort or when analyzed for specific subgroups according to the tumor location (right, transverse, or left colon) or stage of the disease (pT3 or stage III). *Conclusions*: Laparoscopic complete mesocolic excision for colonic cancer can be offered as a standard procedure by experienced surgical teams in carefully selected patients and provides oncological results similar to those obtained with open surgery.

## 1. Introduction

Sharp dissection into embryological planes that provide an en-bloc surgical specimen covered by the intact perirectal fascia is currently recognized as the standard surgical care for rectal cancer. Heald named this technique complete mesorectal resection and demonstrated that it is associated with significantly reduced rates of local recurrence [1]. Applying the same principles for tumors of the colon, Hohenberger advocated for the concept of complete mesocolic excision (CME) and showed similar results of reduced rates of local recurrence and improved long-term survival compared with rectal surgery [2].

Dissection in CME is initiated at the root of the feeding vessels and is then directed from medial to lateral orientations along the fused embryological planes. This preserves intact the fascial cover of the mesocolon and allows for the complete removal of all the lymphatic tissue contained within it, including the central lymph stations. In this approach, the lining fascia of the mesocolon, which represents the most effective barrier against local dissemination of the tumor, is preserved intact and, thus, the risk of intraoperative malignant cell spillage is reduced while the number of lymph nodes retrieved in the surgical specimen is increased. All of these factors prevent the systemic dissemination of tumor cells and improve local control of the disease, leading ultimately to increased rates of survival [3].

Compared to the standard resection technique, CME has been associated with improved overall and disease-free survival in patients with stage I–III adenocarcinoma [4] while in stage III colon cancer, the 5-year loco-regional recurrence rate has also been shown to decrease significantly [5]. Despite these encouraging results and the adoption of CME in centers of excellence, the technique did not become standard care, most probably due to its increased technical complexity, the higher risk of intraoperative and postoperative complications, and a reduced awareness of its advantages in the surgical community. Nowadays, surgery for rectal cancer is centralized, included into multimodal treatment protocols, and standardized to include total mesorectal excision, while colonic resections for cancer are still performed in a wide spectrum of hospitals, by various surgical teams with incomplete credentials for oncologic colorectal surgery and for whom CME may be too difficult or too risky to perform. This situation is ultimately reflected in the overall survival rates for colonic cancer, which, not surprisingly, are now lagging behind those for rectal cancer [6].

The minimally invasive approach to colorectal surgery is currently growing fast, with an ever-increasing number of surgeons attempting to offer this approach to their patients. The short-term outcomes after laparoscopic colonic resection have been proven to be significantly better compared to open surgery, while the long-term survival rates are at least similar [7,8]. However, in patients with advanced disease, such as pT3/4 or stage III colon cancer, there is still ongoing debate as to whether a minimally invasive approach can offer the same quality of dissection in terms of oncological safety as the open technique [9]. It is acknowledged that laparoscopy has certain difficulties in maintaining an optimal view on the dissection plane, especially in obese patients, those with large tumors, and for cancers located in particular segments of the colon such as the transverse colon. Since CME has been proven to be difficult to implement on a large scale in open surgery, there is a legitimate question as to whether a minimally invasive approach to CME is feasible in laparoscopy to the extent of considering it a standard alternative to the open approach.

Our surgical team are enthusiastic proponents of the medial-to-lateral approach of CME for colonic cancer. Over the past 10 years, we have also gained substantial experience in the field of laparoscopic surgery, including colorectal resections [10]. The main aim of this paper is to evaluate whether a laparoscopic CME technique can be considered as standard care in patients with colonic cancer in the hands of experienced teams in minimally invasive colonic surgery. The second objective is to analyze whether Lap-CME offers the same oncological quality of dissection as open surgery.

## 2. Materials and Methods

### 2.1. Data Collection

Clinical data from patients with colon cancer who underwent laparoscopic and open CME at the 1st Surgical Clinic, Emergency County Clinical Hospital of Cluj Napoca, by our surgical team was retrieved from the electronic database of the hospital or from the written medical records and was introduced into an electronic database. The Ethical Commission of the “Iuliu Hatieganu” University of Medicine and Pharmacy granted permission (no. 32/23.03.2023) to retrospectively analyze the data of the patients who were operated on. We confirm that all research was performed in accordance with the Declaration of Helsinki.

All patients with resectable colon cancer were included for analysis. Tumors of the recto-sigmoid junction were considered to be upper rectal cancers and were excluded from the study. In addition, patients who required emergency operations (tumor perforation or bowel obstruction), those with peritoneal carcinomatosis, or systemic (pulmonary) metastasis were also excluded. All patients were investigated preoperatively by a colonoscopy with biopsy for histopathological examination, contrast-enhanced abdominal computed tomography, routine blood tests, and quantitative analysis of specific tumor markers (ACE and CA 19-9).

Allocation of patients into the study groups was not randomized. The decision regarding whether the patient had an indication for laparoscopic or open surgery was taken by the operating surgeon after discussion with the medical team (anesthesiologist, cardiologist) and with the patient. Each patient was initially considered as a candidate for a laparoscopic approach, but a multitude of factors that could influence the final decision towards an open operation were taken into account such as (1) high anesthesia risks (ASA 3 or 4); (2) severe cardiac insufficiency, HYHA III–IV; (3) severe restrictive pulmonary disease; (4) complex previous abdominal operations (a previous laparotomy in itself did not represent an absolute contraindication); (5) low performance status; (6) advanced locoregional disease on preoperative CT scan (suspicion of invasion into the renal fascia, duodenum, bladder, or small bowel, etc.); (7) location of the tumor in the transverse colon (in the first half of the study period); and (8) tumor size above 6 cm, etc. None of these parameters represented an absolute contraindication but, when a combination of them were present, the patient was more likely to be operated on using an open approach. Patients selected for a laparoscopic approach were generally younger, with a higher performance status and fewer cardiopulmonary comorbidities, and were thus more likely to tolerate prolonged periods in the reverse Trendelenburg position. In addition, they usually had smaller, less locally advanced tumors on the preoperative CT scan.

Preoperative workup included mild bowel preparation with lactulose 30 mL administered in three daily doses for the last three days prior to surgery, anticoagulant therapy on the evening before surgery, antibioprophylaxis at the induction of anesthesia followed by three postoperative doses, and the use of intraoperative contention stockings for the prophylaxis of deep vein thrombosis. In obese patients, especially those who were selected for a laparoscopic approach, intraoperative intermittent pressure stockings were also used. Anticoagulant therapy was continued until the 14th postoperative day. Patients were included in the ERAS perioperative protocols with liberal use of peridural anesthesia, early mobilization, early enteral feeding, limited use of intra-abdominal drains, intraoperative removal of the nasogastric tube, and limited postoperative use of opioid analgesia.

### 2.2. Surgical Technique

#### 2.2.1. Right Colectomy with CME Was Used for Tumors Situated in the Cecum and Ascending Colon up to Right Third of the Transverse Colon

This operation can be carried out using an open technique, through a median laparotomy, or by a laparoscopic approach using 5–6 trocars. A medial-to-lateral dissection was used as standard. The visceral peritoneum was incised on the projection line of the superior mesenteric vein (SMV). The SMV was visualized and dissected along its anterior and right lateral border, identifying the origins of the ileocolic and right colic vessels, which were ligated/clipped and divided at origin. The right mesocolon was dissected off the peritoneum along the Told fascia from medial to lateral orientations, exposing the duodenum and the head of the pancreas. The origins of the middle colic vessels were identified, lymphadenectomy was performed along their anterior and right side, and the right branch of the middle colic vessels was ligated/clipped and divided at the origin. The root of the mesocolon was dissected off the pancreatic fascia while carefully identifying and preserving the venous trunk of Henle. The gastro-colic and hepato-colic ligaments were divided close to the stomach/liver followed by transection of the parieto-colic ligament. In open surgery, a manual or stapled latero-lateral anastomosis was performed using the standard procedure. In laparoscopic surgery, an upper transverse mini-laparotomy was used to deliver the specimen and construct the anastomosis before 2016. In the period of 2016–2020, the division of the colon/ileum and the creation of the L-L ileo-transverse anastomosis was performed exclusively using an intracorporeal technique described previously [10], with the specimen being delivered through a Pfannenstiel mini-laparotomy. The mesenteric defect was closed with a laparoscopic running suture.

#### 2.2.2. Transverse Colectomy with CME Was Used for Tumors in the Vicinity of the Middle Third of the Transverse Colon

Transverse colectomy was generally performed by an open approach, but a laparoscopic approach using 5–7 trocars was also used in selected cases. The transverse colon was pushed cranially to open its inferior, visceral part. The peritoneum was incised at the root of the mesentery, on the projection line of the SMV. The SMV and SMA were identified and lymphadenectomy of lymph-node stations no. 214 and no. 223 according to the JSCCR nodal classification was performed [11]. The middle colic artery and vein were ligated/clipped at the origin and divided. The transverse mesocolon was dissected off the pancreatic fascia and divided at an adequate distance from the tumor. The gastrocolic ligament was divided close to the stomach and the omental bursa was entered. The colon was divided proximally and distally with staplers at adequate distances from the tumor. The hepatic and splenic flexures were mobilized, as necessary. The L-L colo-colic anastomosis was performed manually or stapled in open surgery and stapled with intracorporeal sutures in laparoscopic surgery, the specimen being extracted through a Pfannenstiel mini-laparotomy in the latter case. The mesenteric defect was closed with a running suture.

#### 2.2.3. Left Colectomy with CME Was Used for Tumors Located at the Splenic Flexure, and Descending and Sigmoid Colon

Dissection was initiated by incising the visceral peritoneum at the root of the sigmoid mesentery and entering the avascular plane of Toldt. The nervous branches of the superior hypogastric plexus were identified and protected. The origin of the inferior mesenteric artery (IMA) was identified and lymphadenectomy along the IMA up to the level of emergence of the superior rectal artery was performed.

In the case of a tumor of the descending colon, the Riolan arcade and the sigmoid trunk were ligated/clipped and divided at the origin from the IMA. The superior rectal artery was preserved to offer adequate blood supply to the rectal stump. The inferior mesenteric vein (IMV) was divided proximally at the inferior border of the pancreas and distally at the level where it crosses the superior rectal artery. Lymphadenectomy was performed on the anterior and left lateral side of the aorta with preservation of the hypogastric plexus. The descending mesocolon was dissected off of the fascia of Toldt from medial to lateral orientations. The origin of the middle colic artery was identified and lymphadenectomy of station 222 was performed. The left branches of the middle colic vessels were divided at their origin. The splenic flexure of the colon was completely taken down. The colon was divided proximally and distally at the corresponding distances from the tumor. An L-L colo-colic anastomosis was performed using the standard open procedure or the laparoscopic intracorporeal technique. The specimen was removed through a Pfannenstiel incision in laparoscopic surgery.

For tumors of the sigmoid colon, the origin of the IMA was identified and central lymphadenectomy was performed with preservation of the IMA until the emergence of the Riolan arcade, which was preserved. Lymphadenectomy proceeded along the arch of Riolan up to the origin of the left colic artery and along the inferior border of the left colic artery, up to the wall of the left colon. The sigmoid trunk and superior rectal artery were divided at their origin from the IMA. The upper rectum was divided close to the peritoneal reflection. A double-stapled, circular colorectal L-T anastomosis was performed in both the open and laparoscopic approaches. For laparoscopy, the specimen was extracted through a Pfannenstiel mini-laparotomy.

### 2.3. Statistical Analysis

The variables were presented as the median and 25–75 percentiles if continuous and as frequencies and proportions if categorical. The chi-square test was used to compare differences between categorical variables. The Kruskal–Wallis test was performed to analyze the variance of continuous variables. A value of *p* < 0.05 was considered to be statistically significant. Statistical analysis was performed using Medcalc v18.11.6.

#### Calculation of Long-Term Survival

The long-term survival of patients in this study cohort was analyzed by retrieving the data from the National Agency of Inhabitants Welfare. In this setting, we had information pertaining to whether the person was alive or dead at the time of inquiry but did this not provide information about the cause of death. The overall survival was defined as the time that had elapsed between the histopathological confirmation of the tumor and the date of the database inquiry or time of patients’ death, and this was calculated using the Kaplan–Meier method.

## 3. Results

### 3.1. Characteristics of the Study Cohort

In the studied period, 125 patients were diagnosed with colonic neoplasm and underwent surgery by the operating team led by BVV using either an open or laparoscopic approach. Of these, 25 patients did not meet the inclusion criteria. The remaining 100 cases were included in the final analysis.

The mean age of patients from the entire cohort was 66 years (range 34–86 years). Most of the patients had comorbidities classified as ASA 2 (41.8%) or ASA 3 (56.1%) according to the American Society of Anesthesiologists (ASA) score. The most frequent localization of the tumor was in the left colon (49%), followed by the right (40%) and transverse (11%) colon. Most of the tumors were locally advanced: 64% were staged preoperatively as cT3. Stages cT1, cT2, and cT4 accounted for 1%, 7%, and 20% of cases, respectively. Liver metastases were present at diagnosis in 5% of the patients. Approximately one quarter of the patients (24%) had a history of previous laparotomy; of these, 33% underwent a laparoscopic operation and 66% underwent an open approach. Right colectomy was performed in 23 open cases and 16 laparoscopic cases. An extended right colectomy was performed in one case using an open approach and in one case laparoscopically. Left colectomy was performed as an open approach in 23 cases and laparoscopically in 17 cases, while an extended left colectomy was chosen in 4 open cases and 3 laparoscopic cases. Transverse colectomy was predominantly carried out using an open approach (10 cases), while two cases underwent laparoscopic transverse colon resections. Lap-CME was finalized in all patients for whom it was attempted. The general clinical and pathological characteristics of the cohort are presented in Table 1.

### 3.2. Pathological Characteristics of the Surgical Specimen

Pathological assessment of the specimens revealed colonic adenocarcinoma in 86% of cases, in addition to mucinous-type adenocarcinoma (13%) and tubulo-villous adenoma (1%). The mean tumor size for the entire cohort was 4.5 cm (range: 1.3–11.5 cm), 5 cm for the open group and 4 cm for the laparoscopic group. Most of the patients had advanced disease: pT3 tumors were present in 67%, pT4 in 15% (12% = pT4a and 3% = pT4b), pT2 in 10%, and pT1 in only 3% of the cases. Complete resection (R0) was achieved in 98% of patients, with both R1 resections being encountered in cases with large, locally invading tumors where surgery was carried out with an open approach. There was, however, no statistical difference between the two groups in terms of R resection. The mean proximal resection margin was 10.5 cm (range: 1.5–23 cm), the mean distal resection margin was 10 cm (range: 1.5–51 cm), and the mean radial resection margin was 5.5 cm (range: 0.1–15 cm). The mean lymph node count for the entire cohort was 21 (range: 7–49), with an average of 21 nodes being counted in the open group and 20 in the laparoscopic group. Overall, 35% of the patients had lymph node metastases, with 32.7% in the open group (20 of 61 patients) and 38.4% (15 of 39 patients) in the laparoscopic group. The maximum number of positive lymph nodes was 15. The mean metastasized/total lymph nodes ratio was 0.14 for laparoscopic group and 0.09 in open group (*p* = 0.21).

### 3.3. Postoperative Outcome in the Study Cohort

The overall morbidity rate was 20.9%. Among the specific postoperative surgical complications, 4% of the patients had chyloperitoneum, 5% suffered from prolonged postoperative ileus, and 3% developed surgical-site infections. Seven patients required reoperation for complications: two for bleeding, two for peritonitis secondary to anastomotic leak, two for persistent chyloperitoneum, and one patient required negative-pressure wound therapy for a surgical-site infection of the abdominal wound.

The mortality rate was 1%, with one death occurring in the open group in a patient with extensive cardiovascular comorbidities that required anticoagulant therapy and succumbed due to late postoperative hemorrhage. The median time to discharge was 9 days (range: 5–39 days), which was marginally shorter for the laparoscopic group (8 vs. 10 days, *p* = 0.1).

### 3.4. Comparison of the Open vs. Laparoscopic Surgical Groups

The R0 resection rate (*p* = 0.24), length of distal resection margin (*p* = 0.56), proximal resection margin (*p* = 0.15) and radial resection margin (*p* = 0.41), and number of lymph nodes present in the surgical specimen (*p* = 0.488) did not differ significantly between the open and laparoscopic groups.

An evaluation of the type of surgical technique used, open or laparoscopic, depending on allocation of patients in one of the ASA score groups: ASA (I + II) vs. ASA (III + IV), revealed no statistical differences. However, the size of the tumor was significantly larger in the open group (5 cm in the open group vs. 4 cm in the laparoscopy group, *p* = 0.0005).

When the lymph node count and resection margins were analyzed separately for each tumor location and type of surgery, no statistical significance was identified between the open and laparoscopic groups (*p* > 0.05) (Table 2).

When the subgroup of patients with advanced pT3 colonic tumors was analyzed, no significant difference was observed between the open or the laparoscopic groups in terms of the resection margins (*p* > 0.05 for radial, proximal, and distal margins), complete excision rate (*p* = 0.39 for R0 resection rate), and total lymph node count (*p* = 0.43). Similar results were obtained when the subgroup of patients with stage III disease was analyzed. In this latter stage III colonic cancer sub-group, the only significant difference between the open and laparoscopic groups was the radial resection margin (4 cm lap vs. 8 cm open), but this result has no clinical significance since a 4 cm radial resection margin is considered to be sufficient (Table 3).

### 3.5. Long-Term Survival

**Figure 1:**
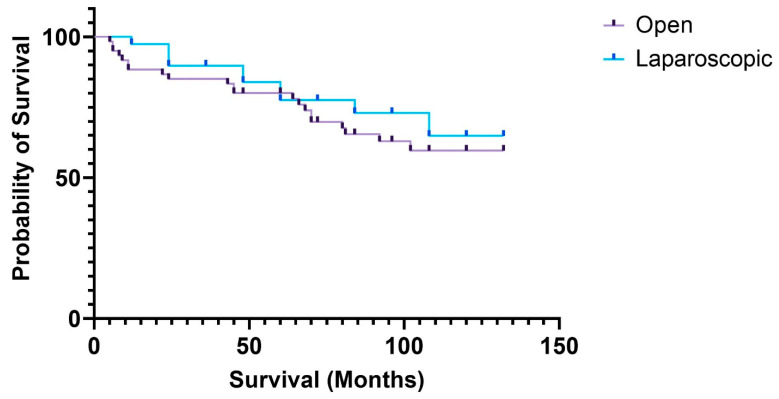
Survival curves of patients with complete mesocolic excision in the open and laparoscopic groups.

The overall survival at 3, 5, and 10 years was 85.89%, 81.58%, and 61.66%, respectively. When compared between the study groups, it averaged 83.33% for the open group vs. 89.74% for the laparoscopic group at 3 years (*p* = 0.864), 80% for the open group vs. 83.95% for the lap group at 5 years (*p* = 0.934), and 59.56% for the open group vs. 64.83% for the lap group at 10 years (*p* = 0.235) (Figure 1) of follow-up.

## 4. Discussion

Colorectal cancer is the third most commonly diagnosed cancer in males and the second in females. Despite recent advancements in surgical technique and the development of sophisticated chemotherapy regimens, significant mortality rates continue to be associated with this disease [12,13].

Apart from the particularities of the surgical technique for each location, three essential components are required to define an adequate CME: (1) the removed mesentery should be intact, contained within a continuous shiny envelope of mesenteric fascia and visceral peritoneum; (2) central vascular ligation (CVL) and removal of all of the regional lymph nodes, including those at the root of the main feeding vessels, are mandatory, and (3) an adequate length of bowel measured longitudinally from the proximal and distal poles of the tumor should be resected in order to remove the corresponding pericolic lymph nodes along the marginal colic vessels [14].

The benefits of CME in open surgery have been proven in the literature. A retrospective study comparing the survival of patients with stage I and II colon cancer between one center that used the CME technique and two centers that performed “standard” colectomy demonstrated an improved 3-year overall survival rate (88.1% vs. 79.0%, *p* = 0.003) and disease-free survival rate (82.1% vs. 74.3%, *p* = 0.026) in the CME group [15]. Another prospective study on patients who underwent surgery for right-sided colon cancer before and after the introduction of CME in a single surgical center showed clear advantages in the local control of the tumor in the CME group (21% recurrence in the control group vs. no local recurrence in the CME group) [16]. It should be noted, however, that a high proportion of patients in these studies were in the early stages of the disease (stage I and II). A recent systematic review of 5246 patients comparing CME with “standard” resection for colon cancer demonstrated significant advantages in terms of the local recurrence rate (4.5% vs. 7.8%), disease-free survival (77.4% vs. 66.7%), and overall survival (58.1% vs. 53.5%) for the patients who underwent CME [17]. Three meta-analyses reported a decrease in local recurrence rates with CME, and one also noted a reduction in distant recurrence. Although CME involves a longer operating time, it results in less intraoperative blood loss and comparable complication rates between CME and standard surgery. Despite concerns about possible vascular, lymphatic, and autonomic injury during CME, studies have consistently found no significant differences in the rates of anastomotic leakage, postoperative mortality, or hospital stay duration compared to conventional colectomy [18].

Several studies have correlated the number of excised lymph nodes with an improved prognosis and increased overall survival, cause-specific survival, and disease-free survival, even for N0 patients, who do not have documented lymph node involvement [3,19]. In our study, the median number of harvested lymph nodes was 21, which is slightly lower compared with other studies from the literature reporting on CME [2,20,21,22]. In this respect, we have to mention that specimens from our study were not treated with fat-clearing techniques during analysis and, thus, small 2–4 mm lymph nodes might have been missed by the pathologist.

The advantages of a minimally invasive approach for the treatment of colonic cancer have already been demonstrated by several randomized controlled trials (COST, MRC CLASSIC) [8,23]. It is associated with decreased pain intensity, reduced postoperative ileus, a lower incidence of surgical-site infections (SSIs), and a shorter hospital stay, all leading to a faster postoperative recovery [6,7]. The available data shows that the long-term oncological outcomes after laparoscopic surgery are also at least comparable, if not superior, to the open approach [24]. However, these trials included patients with both colon and rectal cancer, a significant number of patients were in the early stages of the disease (stage I and II), and the extent of lymphadenectomy was not analyzed according to a systematic design.

Several ongoing randomized trials are comparing CME with central vascular ligation to conventional surgery, but only one has published long-term outcomes so far. In a single-center study, Yan and colleagues randomized 196 patients with stage III colon cancer (left- or right-sided) to receive either laparoscopic CME or standard resection; CME resulted in a higher average lymph node yield (25 vs. 17) and significantly improved disease-free and overall survival. The long-term findings from additional larger multicenter trials, such as the COLD and RELARC trials, may provide more substantial information concerning the benefits of this technique [25].

Our study confirms that reproduction of the complex and difficult CME open technique in a laparoscopic setting is not restricted to a small number of highly selected cases. Although our study cohort was abundant in pT3 tumors, 39% of all patients benefited from a laparoscopic operation without compromising the oncological quality of dissection. When patients with cancer of the transverse colon are excluded, this figure rises to 41.5%. These values could have been even higher but the study design was not randomized and there was some biased selection of patients from the laparoscopic group towards less-advanced cases, although it was not clearly shown by the statistical analysis. This selection was determined by our primary concern to ensure the surgical and oncological safety of the procedure in difficult or marginally difficult cases. In this respect, patients with very large tumors or with obvious loco-regional invasion and cases with significant cardiovascular comorbidities were more likely to undergo surgery using an open technique. Moreover, tumors of the transverse colon, splenic flexure, and proximal part of the descending colon were approached laparoscopically only in the second half of our study, when the surgical team felt better prepared to perform complex dissections and intracorporeal anastomosis. If we narrow the field and analyze only patients with tumors located in the right or left colon with a size of less than 4 cm and an ASA risk of I or II, the proportion of laparoscopic colectomy reaches 64%, proving that, in this population, it has become the standard procedure.

It is also noteworthy that, in this study, Lap-CME was finalized in all patients for whom it was attempted. By using a careful process of patient selection and the aforementioned bias to direct difficult cases to an open approach from the beginning, the need to convert to open surgery or to switch intraoperatively from CME to the standard procedure was avoided.

The second objective of the study was to evaluate the quality of CME in the laparoscopic group compared with the open, control group. Since the surgical specimens were not analyzed in a prospective fashion, we could not calculate the area of the excised mesocolon nor the distance from the tumor to the periphery of the specimen. Instead, we have used the concept of oncological quality of dissection, which included three parameters: (1) the total number of lymph nodes excised; (2) the type of resection (R0, R1, R2), and (3) the value of proximal, distal, and circumferential resection margins. The total number of nodes retrieved with the specimen is recognized as an important independent prognostic factor for survival. Swanson et al. demonstrated an increased overall survival in patients with surgical specimens that have a lymph node count of between 22 and 30 [26]. Moreover, in patients with advanced colon cancer and lymph node metastases, extensive lymph node dissection has been shown to directly improve local control and disease-free survival [3]. In our study, the number of retrieved lymph nodes is consistent with the values advanced by Swanson (overall 23 lymph nodes in the open group vs. 20 in the laparoscopic group, *p* = 0.48) and is not different between the open and laparoscopic groups overall or in the right/transverse/left colon cancer subgroup analyses. The rate of R0 resections, as well as the length of the resection margins, were also not different between the study groups. These results are consistent with the data published by Shin et al., which demonstrated no differences in terms of the length of resection margins or the number of harvested lymph nodes between laparoscopic and open resections for right-colon cancer [27]. A difference was recorded, however, both in the short perioperative outcome, with Lap-CME being associated with improved short-term outcomes including a shorter length of stay and time to solid diet, but also on the long-term outcome, with patients who underwent a laparoscopic operation recording better 5-year disease-free and 5-year overall survival [28,29].

The majority of patients included in comparative studies on CME published in the literature were in the early stages of the disease (stage I and II). Our study is different in this respect, with most of the patients presenting with advanced tumors. When we analyzed the subgroup of stage III patients, we did not find any statistical differences between the parameters obtained in the open and laparoscopic groups. The cohort is still too small to draw definite conclusions but these results are in line with data from the literature [9,30] and demonstrate that, when the selection of patients is meticulous and the surgical team is dedicated, laparoscopy can offer the same quality of radical dissection as open surgery, even in locally advanced cancers. However, when tumor invasion into the adjacent organs is present (small bowel, kidney, etc.), a careful analysis of the technical ability of the surgical team and of the overall duration of the operation is required before attempting a complex minimally invasive resection.

The need for experienced surgical teams as a prerequisite to obtain good results in Lap-CME is confirmed in a study from West et al. that evaluated the quality of surgical specimens after open and laparoscopic colectomy performed by supervised trainees [31]. In this study, the morphometric analysis of the specimens showed similar outcomes for open right- and left-sided CME; however, the lymph node yield was lower for laparoscopic CME compared with open CME and identical to that of non-CME surgery. The authors concluded that, although minimally invasive surgery for colonic tumors is safe and feasible, significant experience is required to achieve the desired level of surgical quality.

The issue of laparoscopic transverse colectomy is still under debate. Gouvas et al. analyzed 90 patients undergoing right, left, and transverse colectomies and showed no differences between the open and laparoscopic CME for locations on the right and left colon. Concerns were raised, however, on the completeness of CME for laparoscopic transverse colectomy, with these specimens showing a reduced distance between the tumor and the point of central vessel ligation, as well as reduced lymph node clearance [32]. Laparoscopic dissection for tumors of the transverse colon is challenging for several reasons: (1) the middle colic vessels need to be identified at their origins from the SMA and SMV, with corresponding risks; (2) both colonic flexures need to be mobilized; and (3) the anastomosis must be performed intracorporeally. All of these features require an experienced surgical team. Several studies published recently report favorable results and advocate for the use of minimally invasive surgery for the radical resections of transverse colon cancer [33,34]. In our case, Lap-CME resection of transverse colonic cancers was performed only in selected patients.

Survival was calculated in our study using the data from the National Agency for the Welfare of Inhabitants and is therefore an overall survival figure and not a cancer-specific one. Therefore, we expect the cancer-specific rates to be higher than those presented in our study. Although the Lap-CME group displayed higher survival rates at 3, 5, and 10-years of follow-up, the differences were not significant. Moreover, we need to take into account that patients who underwent surgery with an open approach had higher ASA scores and an increased incidence of preoperative morbidity, factors that increase the mortality due to causes that are not cancer related.

The main strength of our study is that it included a cohort of patients who were operated on by the same surgical team, using a standardized technique, that was similar both in the open and laparoscopic approaches, offering a small but homogenous study group. The aim of the study was to demonstrate that Lap-CME is not a singular event and can be offered by experienced surgical teams as a standard procedure to selected patients, not necessarily to all patients. In this respect, this study fulfilled its aim.

## 5. Conclusions

Colectomy with CME can be safely performed using a laparoscopic approach in most patients presenting with cancer of the right or left colon. The meticulous selection of patients and careful preoperative preparation are important factors in achieving a successful Lap-CME. When these conditions are fulfilled, experienced surgical teams are able to provide a high level of dissection quality and achieve results that are comparable to the open technique. The same conclusions are valid for cancers of the transverse colon, although this location is still biased more towards an open surgical approach.

## Figures and Tables

**Table 1:** Clinical and pathological baseline characteristics of the patients.

		Open (Number of Cases)	Laparoscopic (Number of Cases)
Age (mean + 95%CI)		66.2097 ± 2.415	63.0938 ± 3.518
Sex	Male	30	19
	Female	31	20
Charlson Comorbidity Index (mean + 95%CI)		6.4561 ± 0.604	5.7391 ± 0.656
ASA score	*1*	0	3
	*2*	23	19
	*3*	36	17
	*4*	2	0
Tumor location	Cecum	7	6
	Ascending colon	15	8
	Transverse colon	19	4
	Descending colon	11	1
	Sigmoid colon	9	20
Average tumor size (cm) (25–75 percentiles)		5	4
		(4–6.5)	(3–4.8)
pT staging		Tis–3	Tis–4
		T1–1	T1–2
		T2–7	T2–6
		T3–39	T3–23
		T4a–7	T4a–4
		T4b–4	T4b–0
Complete R0 resection		100%	96.5%
Resection margin (cm) (25–75 percentiles)	Distal	11	9
		(5.27–14.75)	(5.12–13.37)
	Proximal	11.5	10
		(7.5–17)	(5.12–12.37)
	Radial	6	5
		(3–9.75)	(2.25–7.75)
Average lymph node count Median (min–max)		23	20
		(8–48)	(7–49)
Length of hospital stay (days) (25–75 percentiles)		10	8
		(7–13.25)	(7–11.5)

Note: the total number of cases is 100, therefore the number of patients and percentage values are identical.

**Table 2:** Comparison of oncological safety of resection parameters between the open and laparoscopic groups analyzed for specific locations of the colonic tumors.

	Open	Laparoscopic	*p*-Value
Right colon (40 cases) *Lymph node count* (min–max) *Radial margin* (25–75 percentile)	24 (8–30) 5.5 (2–10)	24 (13–49) 5.5 (3–7)	*p* = 0.631 *p* = 0.702
Transverse colon (11 cases) *Lymph node count* (min–max) *Radial margin* (25–75 percentile)	24 (11–33) 6 (2.5–7)	17 (16–18) 5 (5–5)	*p* = 0.405 *p* = 0.613
Left colon (49 cases) *Lymph* node count (min–max) *Radial margin* (25–75 percentile)	21 (8–40) 6 (3–9.62)	18 (7–47) 4.5 (1–9)	*p* = 0.225 *p* = 0.52

**Table 3:** Comparison of resection margins between the open and laparoscopic groups in patients with stage III disease.

	Open	Laparoscopic	*p*-Value
Radial resection margin (cm) (25–75 percentiles)	8 (5–11)	4 (1–5.5)	0.01
Distal resection margin (cm) (25–75 percentiles)	13 (7.12–15.75)	10 (7–15.25)	0.5
Proximal resection margin (cm) (25–75 percentiles)	10.5 (7–19.5)	10.5 (9.35–12)	0.77
Complete resection (R0)	95%	100%	0.42

## Data Availability

The datasets used and/or analyzed during the current study are available from the corresponding author upon reasonable request.

## References

[1] Heald R.J. (1988). The ‘Holy Plane’ of rectal surgery. J. R. Soc. Med..

[2] Hohenberger W., Weber K., Matzel K., Papadopoulos T., Merkel S. (2009). Standardized surgery for colonic cancer: Complete mesocolic excision and central ligation—Technical notes and outcome. Color. Dis..

[3] Le Voyer T.E., Sigurdson E.R., Hanlon A.L., Mayer R.J., Macdonald J.S., Catalano P.J., Haller D.G. (2003). Colon cancer survival is associated with increasing number of lymph nodes analyzed: A secondary survey of intergroup trial INT-0089. J. Clin. Oncol..

[4] Bertelsen C.A., Neuenschwander A.U., Jansen J.E., Wilhelmsen M., Kirkegaard-Klitbo A., Tenma J.R., Bols B., Ingeholm P., Rasmussen L.A., Jepsen L.V. (2015). Disease-free survival after complete mesocolic excision compared with conventional colon cancer surgery: A retrospective, population-based study. Lancet Oncol..

[5] Merkel S., Weber K., Matzel K.E., Agaimy A., Göhl J., Hohenberger W. (2016). Prognosis of patients with colonic carcinoma before, during and after implementation of complete mesocolic excision. Br. J. Surg..

[6] Brouwer N.P.M., Bos A.C.R.K., Lemmens V.E.P.P., Tanis P.J., Hugen N., Nagtegaal I.D., de Wilt J.H.W., Verhoeven R.H.A. (2018). An overview of 25 years of incidence, treatment and outcome of colorectal cancer patients. Int. J. Cancer.

[7] Fleshman J., Sargent D.J., Green E., Anvari M., Stryker S.J., Beart R.W., Hellinger M., Flanagan R., Peters W., Nelson H. (2007). Laparoscopic colectomy for cancer is not inferior to open surgery based on 5-year data from the COST Study Group trial. Ann. Surg..

[8] Guillou P.J., Quirke P., Thorpe H., Walker J., Jayne D.G., Smith A.M., Heath R.M., Brown J.M., MRC CLASICC Trial Group (2005). Short-term endpoints of conventional versus laparoscopic-assisted surgery in patients with colorectal cancer (MRC CLASICC trial): Multicentre, randomised controlled trial. Lancet.

[9] Kitano S., Inomata M., Mizusawa J., Katayama H., Watanabe M., Yamamoto S., Ito M., Saito S., Fujii S., Konishi F. (2017). Survival outcomes following laparoscopic versus open D3 dissection for stage II or III colon cancer (JCOG0404): A phase 3, randomised controlled trial. Lancet Gastroenterol. Hepatol..

[10] Fagarasan V., Cordos A., Petrisor C., Bintintan A., Chira R., Nickel F., Surlin V., Dindelegan G., Bintintan V. (2020). Which is the Optimal Method of Reconstruction After Laparoscopic Right Hemicolectomy, the Intracorporeal or Extracorporeal Anastomosis Technique?. Chirurgia.

[11] Japanese Society for Cancer of the Colon and Rectum (2010). Japanese Classification of Colorectal Carcinoma.

[12] Bray F., Ferlay J., Soerjomataram I., Siegel R.L., Torre L.A., Jemal A. (2018). Global cancer statistics 2018: GLOBOCAN estimates of incidence and mortality worldwide for 36 cancers in 185 countries. CA Cancer J. Clin..

[13] Torre L., Bray F., Siegel R., Ferlay J., Lortet-Tieulent J., Jemal A. (2015). Global Cancer Statistics, 2012. CA Cancer J. Clin..

[14] Søndenaa K., Quirke P., Hohenberger W., Sugihara K., Kobayashi H., Kessler H., Brown G., Tudyka V., D’Hoore A., Kennedy R.H. (2014). The rationale behind complete mesocolic excision (CME) and a central vascular ligation for colon cancer in open and laparoscopic surgery: Proceedings of a consensus conference. Int. J. Color. Dis..

[15] Storli K.E., Søndenaa K., Furnes B., Nesvik I., Gudlaugsson E., Bukholm I., Eide G.E. (2014). Short term results of complete (D3) vs. standard (D2) mesenteric excision in colon cancer shows improved outcome of complete mesenteric excision in patients with TNM stages I–II. Tech. Coloproctol..

[16] Galizia G., Lieto E., De Vita F., Ferraraccio F., Zamboli A., Mabilia A., Auricchio A., Castellano P., Napolitano V., Orditura M. (2014). Is complete mesocolic excision with central vascular ligation safe and effective in the surgical treatment of right-sided colon cancers? A prospective study. Int. J. Color. Dis..

[17] Killeen S., Mannion M., Devaney A., Winter D.C. (2014). Complete mesocolic resection and extended lymphadenectomy for colon cancer: A systematic review. Color. Dis..

[18] Seow-En I., Chen W.T. (2022). Complete mesocolic excision with central venous ligation/D3 lymphadenectomy for colon cancer—A comprehensive review of the evidence. Surg. Oncol..

[19] Chen S.L., Bilchik A.J. (2006). More extensive nodal dissection improves survival for stages I to III of colon cancer: A population-based study. Ann. Surg..

[20] Hwang D.Y., Lee G.R., Kim J.H., Lee Y.S. (2020). Laparoscopic complete mesocolic excision with D3 lymph node dissection for right colon cancer in elderly patients. Sci. Rep..

[21] Ouyang M., Luo Z., Wu J., Zhang W., Tang S., Lu Y., Hu W., Yao X. (2019). Comparison of outcomes of complete mesocolic excision with conventional radical resection performed by laparoscopic approach for right colon cancer. Cancer Manag. Res..

[22] Reddavid R., Osella G., Evola F., Puca L., Spidalieri L., Rorato L., Sangiuolo F., Solej M., Degiuli M. (2020). Complete mesocolic excision for right colon cancer—State of art: A systematic review of the literature. Ann. Laparosc. Endosc. Surg..

[23] Clinical Outcomes of Surgical Therapy Study Group (2004). A comparison of laparoscopically assisted and open colectomy for colon cancer. N. Engl. J. Med..

[24] Buunen M., Veldkamp R., Hop W.C., Kuhry E., Jeekel J., Haglind E., Påhlman L., Cuesta M.A., Msika S., Colon Cancer Laparoscopic or Open Resection Study Group (2009). Survival after laparoscopic surgery versus open surgery for colon cancer: Long-term outcome of a randomised clinical trial. Lancet Oncol..

[25] Brown K.G.M., Ng K.S., Solomon M.J., Chapuis P.H., Koh C.E., Ahmadi N., Austin K.K.S. (2024). Complete mesocolic excision for colon cancer: Current status and controversies. ANZ J. Surg..

[26] Swanson R.S., Compton C.C., Stewart A.K., Bland K.I. (2003). The prognosis of T3N0 colon cancer is dependent on the number of lymph nodes examined. Ann. Surg. Oncol..

[27] Shin J.K., Kim H.C., Lee W.Y., Yun S.H., Cho Y.B., Huh J.W., Park Y.A., Chun H.-K. (2018). Laparoscopic modified mesocolic excision with central vascular ligation in right-sided colon cancer shows better short- and long-term outcomes compared with the open approach in propensity score analysis. Surg. Endosc..

[28] Bae S.U., Saklani A.P., Lim D.R., Kim D.W., Hur H., Min B.S., Baik S.H., Lee K.Y., Kim N.K. (2014). Laparoscopic-assisted versus open complete mesocolic excision and central vascular ligation for right-sided colon cancer. Ann. Surg. Oncol..

[29] Huang J.L., Wei H.B., Fang J.F., Zheng Z.H., Chen T.F., Wei B., Huang Y., Liu J.P. (2015). Comparison of laparoscopic versus open complete mesocolic excision for right colon cancer. Int. J. Surg..

[30] Zheng Z., Jemal A., Lin C.C., Hu C.Y., Chang G.J. (2015). Comparative effectiveness of laparoscopy vs open colectomy among nonmetastatic colon cancer patients: An analysis using the National Cancer Data Base. J. Natl. Cancer Inst..

[31] West N.P., Kennedy R.H., Magro T., Luglio G., Sala S., Jenkins J.T., Quirke P. (2014). Morphometric analysis and lymph node yield in laparoscopic complete mesocolic excision performed by supervised trainees. Br. J. Surg..

[32] Gouvas N., Pechlivanides G., Zervakis N., Kafousi M., Xynos E. (2012). Complete mesocolic excision in colon cancer surgery: A comparison between open and laparoscopic approach. Color. Dis..

[33] Zmora O., Bar-Dayan A., Khaikin M., Lebeydev A., Shabtai M., Ayalon A., Rosin D. (2010). Laparoscopic colectomy for transverse colon carcinoma. Tech. Coloproctol..

[34] Zhao L., Wang Y., Liu H., Chen H., Deng H., Yu J., Xue Q., Li G. (2014). Long-Term Outcomes of Laparoscopic Surgery for Advanced Transverse Colon Cancer. J. Gastrointest. Surg..

